# Electronic Glycemic Management Systems Versus Conventional Insulin Infusion Protocols in Diabetic Ketoacidosis: A Systematic Review and Meta-Analysis of Non-Randomized Studies

**DOI:** 10.3390/medicina62071287

**Published:** 2026-07-03

**Authors:** Adnan Bhat, Abdullah, Asad Zaman, Ali Shan Hafeez, Muhammad Faizan, Abdul Rafae Faisal, Muhammad Asad, Syed Zaeem Ahmed, Shaikh Muhammad Daniyal, Arkadeep Dhali, Juan M. Munoz Pena

**Affiliations:** 1 Department of Hospital Medicine, University of Florida, Gainesville, FL 32611, USA; adnaen@gmail.com; 2 Department of Medicine, Rawalpindi Medical University, Rawalpindi 46000, Punjab, Pakistan; abdulahabid6@gmail.com; 3 CMH Multan Institute of Medical Science, Multan 53400, Punjab, Pakistan; zamanasad386@gmail.com (A.Z.); alishanhaffeez@gmail.com (A.S.H.); abdulrafaefaisal123@gmail.com (A.R.F.); 4 Hamad Medical Corporation, Doha P.O. Box 3050, Qatar; mfaizanyousuf98@gmail.com; 5 Dow Institute of Health Sciences, Karachi 74200, Sindh, Pakistan; m.asadresearch@gmail.com (M.A.); syed.zaeemahmed@outlook.com (S.Z.A.); daniyal.shk2003@gmail.com (S.M.D.); 6 School of Medicine, Dentistry, and Biomedical Sciences, Queen’s University Belfast, Belfast BT7 1NN, UK; 7 Division of Endocrinology, Diabetes & Metabolism, University of Florida, Gainesville, FL 32611, USA; munozpenaj@ufl.edu

**Keywords:** diabetic ketoacidosis, electronic glycemic management system, computer-guided insulin therapy, intravenous insulin infusion, systematic review

## Abstract

*Background and Objectives*: Electronic glycemic management systems (eGMSs) are increasingly used to guide intravenous insulin infusion for hospitalized patients with diabetic ketoacidosis (DKA), but available comparative evidence remains non-randomized and clinically heterogeneous and includes multiple algorithmically distinct platforms. *Methods and Materials*: We conducted a systematic review and meta-analysis in accordance with PRISMA guidance and a prospectively registered protocol (PROSPERO: 2025 CRD420251019614). Seven non-randomized studies comprising 3874 hospitalized patients were included; six studies contributed data to the primary meta-analysis of time to DKA resolution. The primary outcome was time to DKA resolution. Secondary outcomes included ICU length of stay (LOS), hospital LOS, duration of insulin infusion, and hypoglycemia (mild and severe). Random-effects models were applied. *Results*: Six studies contributed to the primary meta-analysis of time to DKA resolution; across the review, seven studies included 3874 patients. The pooled analysis showed no statistically significant difference between groups (SMD −0.04, 95% CI −0.22 to 0.14; I^2^ = 74.8%). Recorded hypoglycemia was lower among patients managed with eGMS; however, these estimates should be interpreted cautiously because of heterogeneity, serious-to-critical risk of bias, and potential differences in glucose-monitoring and documentation practices. ICU and hospital LOSs and duration of insulin infusion showed no statistically significant differences overall, with heterogeneity across studies. *Conclusions*: In available non-randomized evidence, eGMS-guided insulin infusion was not associated with a clear difference in time to DKA resolution, ICU length of stay, hospital length of stay, or insulin infusion duration compared with conventional protocols. Lower recorded hypoglycemia was observed with eGMSs, but this finding should be considered hypothesis-generating because of serious-to-critical risk of bias, residual confounding, substantial heterogeneity, and possible detection bias. High-quality randomized trials or target trial emulation studies are needed before recommending widespread adoption, as certainty of evidence was very low across all outcomes.

## 1. Introduction

Diabetic ketoacidosis (DKA) is an acute, life-threatening metabolic emergency that requires prompt recognition and management. DKA typically affects individuals with type 1 diabetes mellitus (T1DM) and is characterized by absolute insulin deficiency, leading to hyperglycemia, ketonemia, and/or ketonuria and high-anion-gap metabolic acidosis [1,2,3]. The reported incidence of DKA in adults with T1DM varies across Europe, the U.S., and Israel, ranging from 0 to 56 events per 1000 person-years [4]. Although DKA is classically associated with T1DM, it can also occur in patients with type 2 diabetes mellitus (T2DM), particularly during acute illness, infection, insulin omission, or severe metabolic stress, and it may overlap clinically with a hyperosmolar hyperglycemic state (HHS) in hospitalized populations. In developing countries, DKA episodes affect 3.8–73.4% of the diabetes population [5]. This substantially burdens healthcare systems, contributing to nearly half a million hospital days annually and incurring approximately $2.4 billion USD in direct and indirect costs [6], against a broader national diabetes burden described in surveillance reports [7]. Inappropriate management of DKA treatment can lead to prolonged hyperglycemia or increased risk of hypoglycemia [8]. Studies have shown that dysglycemia and hypoglycemia in critically ill patients are associated with worse clinical outcomes [8]. Thus, it is imperative to control hyperglycemia in DKA.

Continuous intravenous insulin infusion (CII) is widely accepted as the standard of care for treating patients with DKA [9,10]. Various conventional protocols and eGMS algorithms have been used to manage inpatient hyperglycemia [11,12]. Historically, paper-based protocols (PBPs) were utilized to determine insulin infusion rates and rate changes as needed for glucose emergencies. Paper-based protocols use weight-based dosing to determine the initial insulin infusion rate. However, in recent years, some hospitals have moved away from PBPs in favor of electronic glycemic management systems (eGMSs) for treating and managing DKA. eGMSs adjust insulin doses by considering different patient factors, including height, weight, age, sex, serum creatinine, steroid dosing, renal function, diabetes status, insulin response, and estimated residual extracellular insulin [13].

Although eGMS platforms are often grouped together as computerized insulin-guidance systems, they are not identical interventions. Platforms such as Glucommander, EndoTool, Glytec, GlucoStabilizer, and Accu-Chek IV infusion differ in algorithm design, insulin-adjustment logic, monitoring requirements, alert systems, and integration with local clinical workflows. Therefore, pooling these systems assumes a shared intervention class based on computerized insulin titration rather than identical therapeutic mechanisms.

Individual studies have suggested that eGMSs may improve selected aspects of glycemic safety, particularly hypoglycemia prevention, but comparative evidence in DKA remains limited, heterogeneous, and largely observational. Given the increasing adoption of eGMSs in hospitals and the critical need for optimized DKA management, a comprehensive synthesis of existing evidence is warranted. Adult and pediatric DKA populations were included because both may be treated with intravenous insulin infusion protocols, and both may be exposed to eGMSs in acute care settings. However, age-related differences in insulin sensitivity, DKA severity, cerebral edema risk, and management strategies may modify treatment effects. Age group was therefore treated as an important source of clinical heterogeneity. This study aimed to systematically review and meta-analyze available comparative evidence evaluating eGMSs versus conventional insulin infusion protocols in hospitalized patients with DKA, with particular attention to time to DKA resolution, hypoglycemia, length of stay, heterogeneity across platforms and populations, and certainty of evidence.

## 2. Materials and Methods

The systematic review and meta-analysis were conducted in accordance with PRISMA guidance [14] and MOOSE guidelines [15]. The completed MOOSE checklist is provided as Supplementary Table S1. Because this review used previously published aggregate data, patient consent and institutional review board approval were not required. The study protocol was developed by the authors and registered on PROSPERO on 30 March 2025, before final data extraction and quantitative synthesis, under registration ID CRD420251019614.

### 2.1. PICO (Population, Intervention, Comparison and Outcome) Tool

The PICO framework was used to formulate the clinical research question (Table 1).

### 2.2. Aim and Outcomes

The primary outcome was time to resolution of DKA. DKA resolution definitions were extracted as reported in each study, including criteria based on anion gap, bicarbonate, pH, blood glucose, or combinations of these measures. Because definitions varied across studies, study-level diagnostic and resolution criteria are summarized in Table 2, and variation in definitions was considered a major source of clinical heterogeneity. The secondary outcomes are detailed in Table 3. Where data allowed, sensitivity analysis was planned for studies using standardized DKA resolution criteria. When too few studies used comparable definitions, findings were summarized narratively rather than interpreted as definitive pooled effects.

### 2.3. Search Strategy

A comprehensive literature search was conducted on PubMed, Embase, Scopus, Cochrane Central Register of Controlled Trials, and ClinicalTrials.gov, from database inception to 30 March 2025. The search strategy combined controlled vocabulary terms and free-text keywords related to diabetic ketoacidosis, hyperglycemic crises, computerized insulin infusion, electronic glycemic management systems, and named eGMS platforms. No restrictions were applied based on study design. Only English-language full-text articles were included because translation resources were unavailable; this restriction was considered a potential source of language and selection bias. The final search was conducted on 30 March 2025. Database sources, search date, and record counts are reported in the Methods and PRISMA flow diagram. The full search strings have been provided in Supplementary Table S2.

#### Study Selection

All records identified through database searching were imported into a reference management software, and duplicates were removed. Two reviewers independently screened titles and abstracts for eligibility after piloting the eligibility criteria on a sample of records to ensure consistent interpretation. Full-text articles were then retrieved and assessed independently for inclusion according to predefined eligibility criteria. Disagreements at any stage were resolved through discussion and consensus, with adjudication by a third reviewer when consensus could not be reached. Inter-rater reliability was not formally quantified and is acknowledged as a methodological limitation.

Studies were included if they evaluated adult or pediatric patients with diabetic ketoacidosis and compared a computer-guided or electronic insulin infusion system with a conventional, provider-directed or paper-based insulin infusion protocol. Studies without a comparator group, non-human studies, and studies not reporting relevant clinical outcomes were excluded. The study selection process is summarized in a PRISMA 2020 flow diagram. The initial database search yielded 966 records, of which 189 duplicates were removed.

### 2.4. Inclusion and Exclusion Criteria

Studies were considered eligible if they met the inclusion criteria listed in Table 3. Studies were excluded according to the exclusion criteria listed in Table 3.

### 2.5. Data Extraction

Data extraction was based on key details from included studies using a standardized extraction sheet. Extracted information included study identifiers, year of publication, study design, setting, population characteristics, DKA diagnostic criteria, DKA resolution criteria, DKA severity, eGMS platform, comparator protocol, glucose-monitoring frequency, protocol adherence, adjusted and unadjusted estimates where available, duration of follow-up, and clinical outcomes. The primary outcome was time to DKA resolution. Secondary outcomes were ICU length of stay, hospital length of stay, duration of insulin infusion, and mild and severe hypoglycemia. Disagreements during extraction were resolved through discussion and consensus, or by a third reviewer if necessary.

### 2.6. Risk of Bias Analysis

Risk of bias for non-randomized studies was assessed using the ROBINS-I Version 2 tool, which evaluates seven domains of potential bias, including confounding, selection of participants, classification of interventions, deviations from intended interventions, missing data, outcome measurement, and selection of reported results. Assessments were performed independently by two reviewers, with all disagreements resolved through discussion and consensus. Each study was assigned an overall judgment of low, moderate, serious, or critical risk of bias according to ROBINS-I V2 criteria. Overall risk of bias was determined by the highest risk rating across domains, in accordance with ROBINS-I guidance.

Because ROBINS-I evaluates risk of bias relative to a hypothetical target trial, domains related to confounding, participant selection, intervention classification, deviations from intended interventions, missing data, outcome measurement, and selective reporting were interpreted in relation to the question of whether eGMSs would have differed from conventional insulin infusion under comparable baseline eligibility, treatment assignment, follow-up, and outcome-measurement conditions. Certainty of evidence for each primary and secondary outcome was assessed using the GRADE framework and summarized in a GRADE evidence profile (Table 4).

### 2.7. Data Analysis

Continuous outcomes were synthesized using mean differences (MDs) when outcomes were reported on the same clinical scale, such as hours. Standardized mean differences (SMDs) were used only when studies reported conceptually similar outcomes using non-comparable measurement approaches. Dichotomous outcomes were pooled as odds ratios (ORs). Because time to DKA resolution was reported in hours but defined differently across studies, the MD in hours was prioritized for clinical interpretability where possible, and the impact of differing resolution definitions was evaluated through sensitivity or narrative analysis. Given anticipated clinical and methodological heterogeneity, we used random-effects meta-analysis. The between-study variance (τ^2^) was estimated using restricted maximum likelihood (REML). Because several outcomes included only a small number of studies, Hartung–Knapp–Sidik–Jonkman (HKSJ) confidence intervals were calculated in addition to conventional random-effects confidence intervals. HKSJ-adjusted analyses were used as sensitivity analyses to assess the robustness of inference from conventional random-effects models, and the small number of studies per outcome was considered when interpreting precision. All primary meta-analyses were conducted in Stata (version 19), with REML estimation and HKSJ-adjusted inference implemented in R using the metafor package. Heterogeneity was assessed using Cochran’s Q and the I^2^ statistic, and sensitivity analyses (leave-one-out) were performed to evaluate the influence of individual studies. Prespecified subgroup analyses included adult versus pediatric population, ICU versus non-ICU setting, DKA severity, eGMS platform type, study design, and DKA-only versus mixed DKA/HHS cohorts, where at least two studies were available per subgroup. Meta-regression was not performed because the number of studies per outcome was too small for reliable study-level regression. Most pooled analyses used unadjusted aggregate outcome data because adjusted estimates were inconsistently reported. Adjusted estimates, when available, were extracted and summarized narratively. The use of unadjusted or variably adjusted estimates was considered a methodological limitation. HKSJ-adjusted estimates were used to assess inferential robustness, but because the number of studies per outcome was small, these analyses were interpreted cautiously and were not treated as definitive.

## 3. Results

### 3.1. Search Results and Study Selection

**Figure 1:**
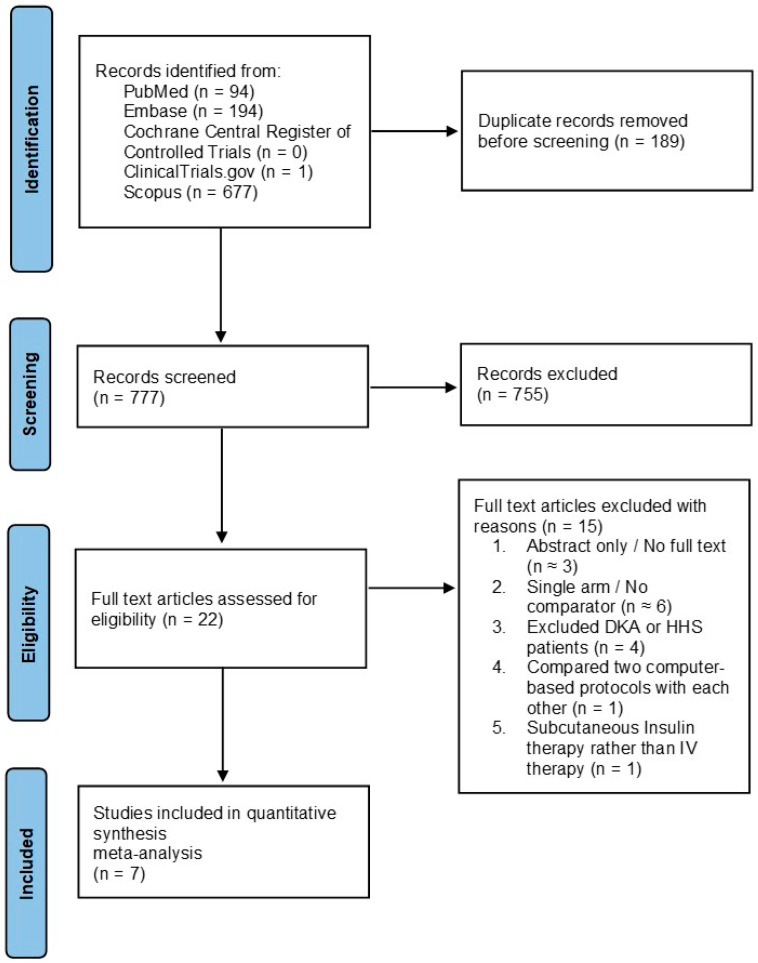
PRISMA flow diagram of study selection.

A total of 966 studies were identified through PubMed (94), Embase (194), Scopus (677), ClinicalTrials.gov (1), and the Cochrane Central Register of Controlled Trials (0). After removing 189 duplicates, 755 studies were excluded for not meeting the inclusion criteria. Twenty-two studies underwent full-text screening, of which 15 were excluded, leaving seven non-randomized studies for final inclusion in the systematic review; six studies contributed data to the primary meta-analysis of time to DKA resolution (Figure 1).

### 3.2. Characteristics of Included Studies

Seven non-randomized observational studies comprising 3874 patients were included in the systematic review, including retrospective cohort, retrospective chart review, and retrospective before–after designs. Included studies evaluated hospitalized patients treated for DKA in ICU, emergency department, or step-down settings. Intervention platforms included Glucommander, Glytec, EndoTool, GlucoStabilizer, and Accu-Chek IV infusion, while comparator groups received conventional paper-based, column-based, provider-directed, or institutional insulin infusion protocols. Reporting of baseline characteristics varied across studies; age and baseline glycemic measures were commonly reported, whereas BMI, APACHE II score, DKA severity, and protocol-adherence details were inconsistently available. Complete baseline characteristics of the included studies are presented in Table 5.

Additional study-level details relevant to clinical and methodological heterogeneity are summarized in Table 2, Table 6 and Table 7, and certainty of evidence is summarized in Table 4. These include DKA diagnostic and resolution definitions, hypoglycemia thresholds, eGMS platform characteristics, glucose-monitoring procedures, protocol adherence, and adjustment approaches. Information not reported in the original study files was recorded as NR.

### 3.3. Risk of Bias Assessment

**Figure 2:**
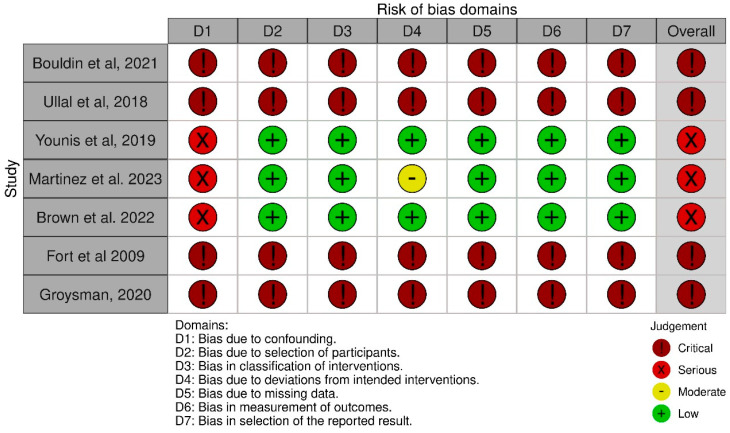
Traffic light plot using ROBINS-I V2 tool [16,17,18,19,20,21,22].

Risk of bias was assessed using the ROBINS-I tool (version 2) across seven domains independently by two reviewers according to prespecified criteria. Overall, the included non-randomized studies were judged to be at serious to critical risk of bias (Figure 2). The primary contributors to elevated risk were confounding and selection of participants, particularly among retrospective cohort studies.

Bias due to confounding was the most prominent concern. Four retrospective studies (Bouldin, Fort, Groysman, and Ullal) were judged at critical risk in this domain because no or inadequate adjustment was performed for key prognostic factors that would have been balanced in a target randomized trial, including baseline DKA severity, comorbidities, diabetes type, and infection status. The remaining studies were judged at serious risk where they provided comparatively clearer intervention definitions, outcome ascertainment, or adjustment approaches, although none were judged to be at low or moderate overall risk of bias.

Bias in the classification of interventions was also more pronounced in retrospective studies (Bouldin, Fort, Groysman, and Ullal), in which treatment assignment reflected routine clinical practice and intervention status was determined from medical records rather than prespecified protocols, resulting in critical risk. Studies with clearer descriptions of intervention assignment and treatment protocols were judged at lower risk in this domain, although the overall risk of bias remained serious.

Substantial variability was observed in bias related to participant selection. The retrospective studies (Bouldin, Fort, Groysman, and Ullal) were judged at critical risk due to potential immortal time bias and selection based on post-intervention characteristics. In contrast, studies with clearer eligibility criteria and better alignment between treatment initiation and follow-up were judged at lower domain-level risk, although the overall risk of bias remained serious.

Bias due to deviations from intended interventions was lower in studies with clearer protocol descriptions, but protocol adherence, treatment deviations, and crossover information were not consistently reported across the evidence base. Retrospective studies (Bouldin, Fort, Groysman, and Ullal) were judged at critical risk owing to inconsistent protocol adherence and limited documentation of treatment changes.

Missing data posed concerns across several studies, particularly retrospective cohorts (Bouldin, Fort, Groysman, and Ullal) with incomplete charting and missing laboratory values, resulting in critical risk. Studies with more complete reporting and clearer data collection procedures were judged at lower risk for missing data, although the overall risk of bias remained serious or critical.

Outcome measurement was largely objective, relying on laboratory-defined endpoints such as DKA resolution and blood glucose levels. Studies with clearer outcome definitions and more complete outcome ascertainment were judged at lower risk in this domain, whereas studies with inconsistent measurement timing or incomplete documentation were judged at higher risk.

Finally, bias in the selection of reported results was judged to be critical in several retrospective studies (Bouldin, Fort, Groysman, and Ullal) due to incomplete reporting and absence of prespecified statistical analysis plans, while other studies had lower concerns in this domain but still had a serious overall risk of bias.

Overall, four retrospective studies (Bouldin, Fort, Groysman, and Ullal) were rated at critical overall risk of bias, primarily driven by confounding and participant-selection bias. The remaining studies were rated at a serious overall risk of bias. No study met the criteria for an overall low or moderate risk of bias. Therefore, all pooled estimates should be interpreted as low- or very low-certainty observational associations rather than evidence of causal treatment effects. Protocol adherence rates, crossovers, deviations from intended insulin protocols, and whether analyses followed intention-to-treat, as-treated, or per-protocol principles were not consistently reported across studies (Table 7). Because non-adherence could not be consistently accounted for, pooled estimates should be interpreted as aggregate observational estimates rather than intention-to-treat causal effects. Certainty of evidence was rated very low for all outcomes using GRADE, primarily because of serious-to-critical risk of bias, inconsistency, indirectness, and imprecision (Table 4).

### 3.4. Primary Outcome: Time to DKA Resolution

**Figure 3:**
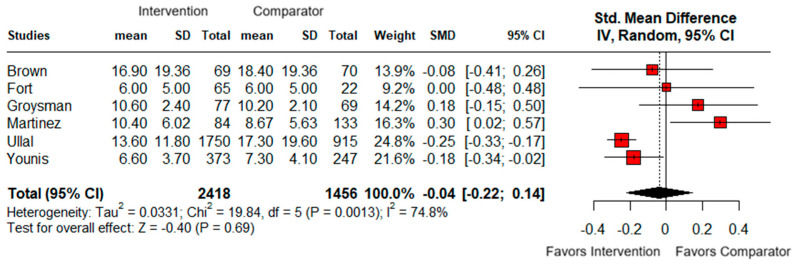
Forest plot of time to DKA resolution [17,18,19,20,21,22].

Six studies evaluated time to DKA resolution; however, definitions of DKA resolution varied across studies. The pooled analysis did not demonstrate a statistically significant difference between groups (SMD −0.04, 95% CI −0.22 to 0.14; *p* = 0.69), but substantial heterogeneity was present (I^2^ = 74.8%), limiting interpretability (Figure 3). Study-level DKA diagnostic and resolution definitions are presented in Table 8. Because resolution definitions differed across studies, including the use of anion gap, bicarbonate, blood glucose, acidosis correction, or transition-related criteria, the pooled estimate should be interpreted cautiously and should not be considered evidence of equivalence between interventions.

**Figure 4:**
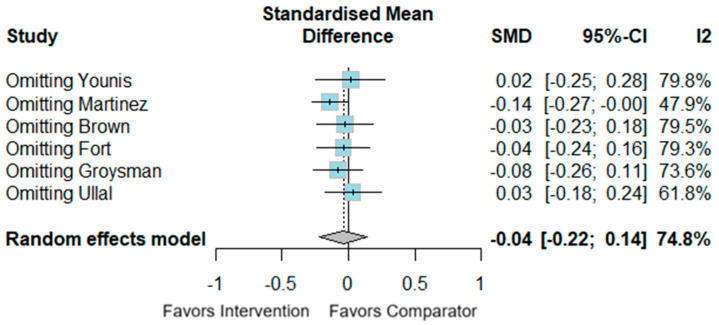
Sensitivity analysis of time to DKA resolution [17,18,19,20,21,22].

Leave-one-out sensitivity analysis did not adequately explain the heterogeneity, suggesting that variability in DKA resolution definitions, baseline severity, clinical setting, and eGMS platform may have contributed to inconsistent effects (Figure 4).

**Figure 5:**
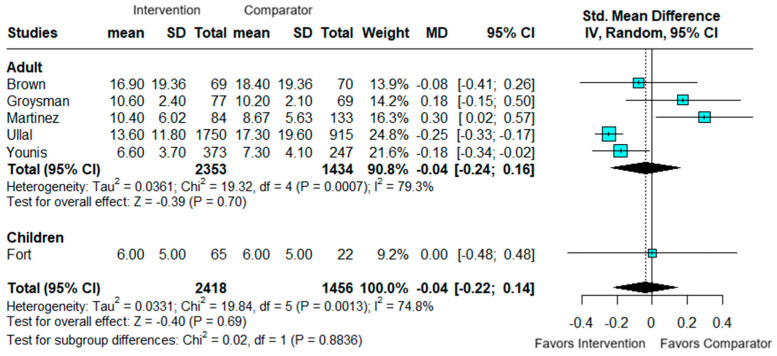
Subgroup analysis of time to DKA resolution [17,18,19,20,22].

Adult-versus-pediatric subgroup analysis did not identify a statistically significant subgroup difference (*p* = 0.88), although the analysis was limited by the small number of studies and should not be interpreted as evidence that age does not modify treatment effect (Figure 5). Planned subgroup analyses by DKA severity, ICU versus non-ICU setting, study design, and eGMS platform were limited by sparse data and the small number of studies per subgroup. Therefore, these factors were explored narratively rather than by formal meta-regression.

### 3.5. Secondary Outcomes

#### 3.5.1. ICU LOS (Intensive Care Unit Length of Stay)

**Figure 6:**
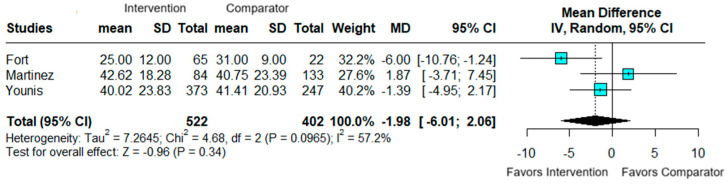
Forest plot of ICU LOS (intensive care unit length of stay) [18,19,21].

Three studies involving 924 patients (522 eGMSs vs. 402 conventional protocols) assessed ICU LOS. The pooled results did not demonstrate a statistically significant difference in ICU length of stay between groups (MD −1.98 h; 95% CI −6.01 to 2.06; *p* = 0.34), with moderate heterogeneity (I^2^ = 57.2%) (Figure 6).

**Figure 7:**
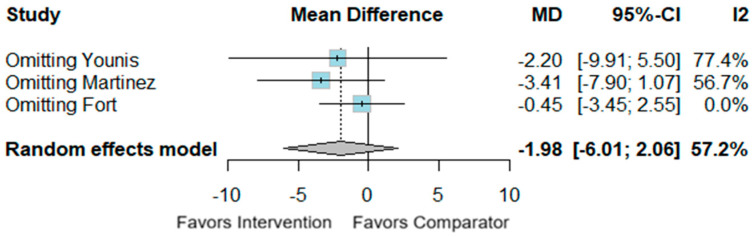
Sensitivity analysis of ICU LOS (intensive care unit length of stay) [18,19,21].

Leave-one-out sensitivity analysis identified Fort et al. as an important contributor to heterogeneity; after its exclusion, I^2^ decreased to 0%, but the pooled estimate remained compatible with no difference between groups (MD −0.45 h; 95% CI −3.45 to 2.55) (Figure 7).

#### 3.5.2. Hospital LOS (Length of Stay)

**Figure 8:**
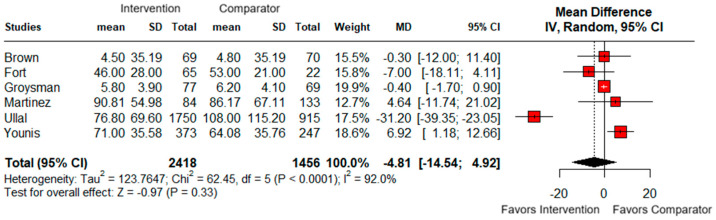
Forest plot hospital LOS (length of stay) [17,18,19,20,21,22].

Five studies including 3874 patients (2418 eGMSs vs. 1456 conventional protocols) assessed hospital LOS. The pooled results did not demonstrate a statistically significant difference in hospital length of stay between groups (MD −4.81 h; 95% CI −14.54 to 4.92; *p* = 0.33), with substantial heterogeneity (I^2^ = 92%) (Figure 8).

**Figure 9:**
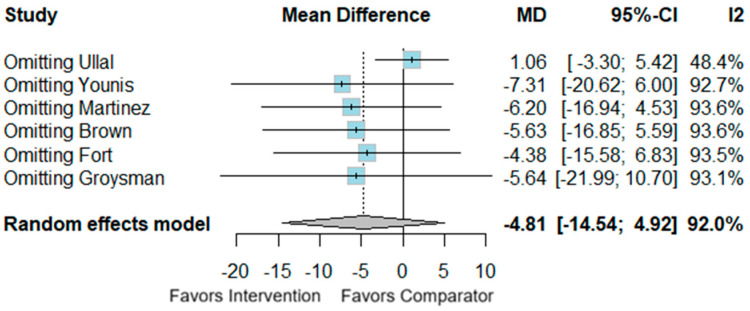
Sensitivity analysis of hospital LOS (length of stay) [17,18,19,20,21,22].

Leave-one-out sensitivity analysis identified Ullal et al. as an important contributor to heterogeneity; its exclusion reduced I^2^ from 92% to 48.4% and changed the direction of the point estimate (MD 1.06 h; 95% CI −3.30 to 5.42), indicating that no directional conclusion regarding hospital length of stay should be inferred (Figure 9).

#### 3.5.3. Duration of Insulin Infusion

**Figure 10:**
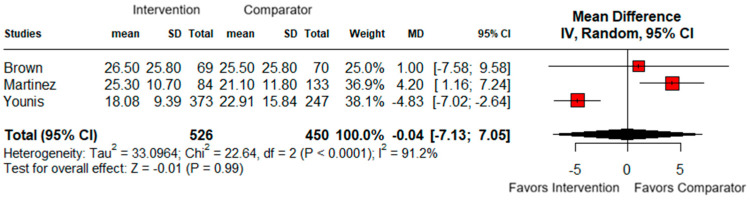
Duration of insulin infusion [18,19,20].

Three studies with 976 patients (526 eGMSs vs. 450 conventional protocols) evaluated the duration of insulin infusion. Studies evaluating the duration of insulin infusion showed highly inconsistent findings, with extreme heterogeneity (I^2^ = 91.2%); therefore, this outcome was considered unsuitable for definitive pooled interpretation (Figure 10).

**Figure 11:**
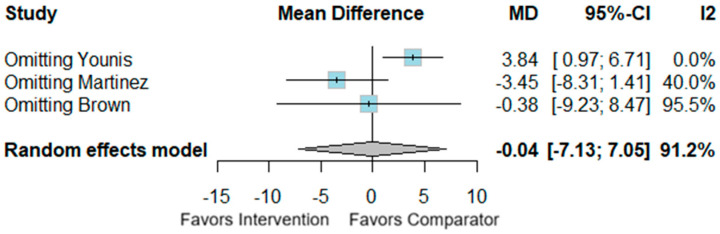
Sensitivity analysis of duration of insulin infusion [18,19,20].

Excluding Younis et al. reduced I^2^ to 0% and changed the estimate to an MD of 3.84 h (95% CI 0.97 to 6.71), indicating that the result was highly sensitive to a single study. This outcome is therefore summarized narratively rather than interpreted as a robust pooled effect (Figure 11).

#### 3.5.4. Mild Hypoglycemia

Six studies, including 3790 patients (2371 eGMSs vs. 1419 conventional protocols), evaluated recorded mild hypoglycemia; however, hypoglycemia thresholds varied across studies and are summarized in Table 5.

**Figure 12:**
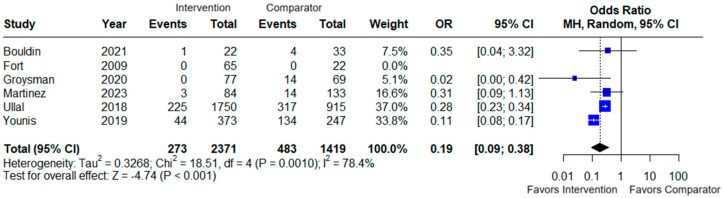
Mild hypoglycemia [16,17,18,19,21,22].

Recorded mild hypoglycemia was lower among patients managed with eGMSs than among those managed with conventional protocols (OR 0.19; 95% CI 0.09 to 0.38), but substantial heterogeneity was present (I^2^ = 78.4%), and the estimate may be affected by residual confounding and differential glucose-monitoring practices (Figure 12).

**Figure 13:**
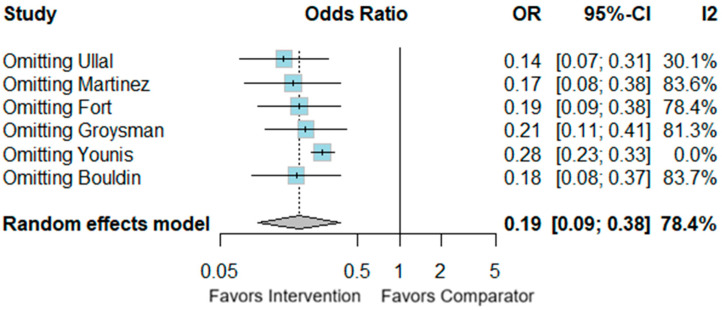
Sensitivity analysis of mild hypoglycemia [16,17,18,19,21,22].

Sensitivity analysis identified Younis et al. as an important contributor to heterogeneity; excluding this study reduced I^2^ to 0% and yielded an OR of 0.28 (95% CI 0.23 to 0.33), indicating that the pooled estimate was influenced by study-level differences (Figure 13).

#### 3.5.5. Severe Hypoglycemia

Six studies, including 3790 patients (2371 eGMSs vs. 1419 conventional protocols), evaluated recorded severe hypoglycemia; however, severe hypoglycemia thresholds varied across studies and are summarized in Table 5.

**Figure 14:**
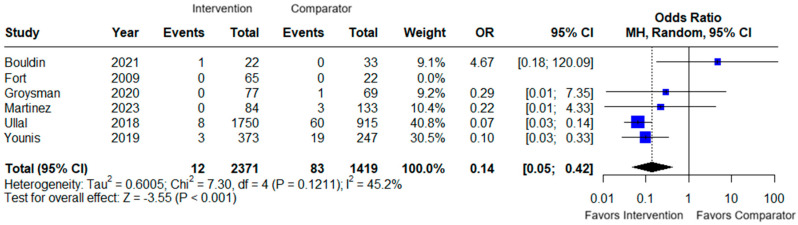
Severe hypoglycemia [16,17,18,19,21,22].

Recorded severe hypoglycemia was lower among patients managed with eGMSs than among those managed with conventional protocols (OR 0.14; 95% CI 0.05 to 0.42), with moderate heterogeneity (I^2^ = 45.2%) (Figure 14).

**Figure 15:**
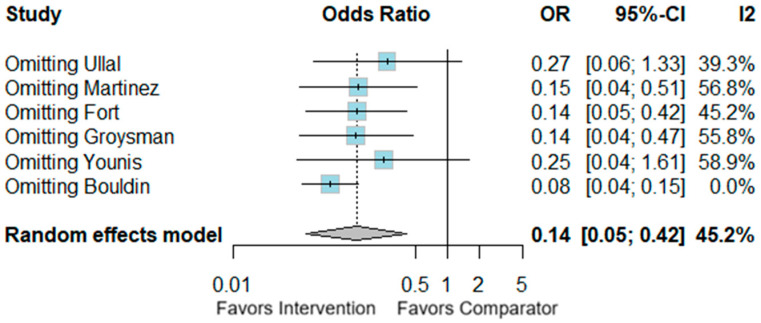
Sensitivity analysis of severe hypoglycemia [16,17,18,19,21,22].

Leave-one-out sensitivity analysis showed that exclusion of Bouldin et al. reduced I^2^ to 0% and changed the pooled estimate to an OR of 0.08 (95% CI 0.04 to 0.15), indicating the sensitivity of the result to a single study. Differences in hypoglycemia thresholds, monitoring frequency, population mix, protocol adherence, or documentation practices may explain this influence (Figure 15).

## 4. Discussion

Several non-randomized studies have evaluated the efficacy and safety of electronic glycemic management systems (eGMSs) compared with conventional insulin infusion protocols in managing diabetic ketoacidosis (DKA) among hospitalized patients. However, to date, no randomized controlled trial has directly compared eGMSs with conventional protocols in DKA, limiting the ability to determine the causal superiority of any specific system. To address this gap in the literature, our meta-analysis pooled available non-randomized evidence to provide a comprehensive assessment of eGMSs compared with conventional protocols across multiple clinical outcomes.

For the primary efficacy outcome, the pooled analysis did not demonstrate a clear difference in time to DKA resolution between eGMSs and conventional protocols. ICU length of stay, hospital length of stay, and duration of insulin infusion also did not show robust evidence of benefit with eGMSs. Lower recorded hypoglycemia was observed in several studies, but this finding must be interpreted alongside serious-to-critical risk of bias, heterogeneous hypoglycemia definitions, and variation in glucose-monitoring and documentation practices [16,17,18,19,20,21,22]. These findings suggest a potential hypoglycemia safety signal with eGMSs, but the current evidence is insufficient to support broad implementation recommendations over conventional protocols.

Among the seven included studies in this meta-analysis, all investigated the efficacy or safety of eGMSs compared with conventional insulin infusion protocols in hospitalized patients with DKA. While the overall pooled results showed no statistically significant difference in time to DKA resolution, individual studies reported some variable findings. Martinez et al. observed similar DKA resolution times between eGMSs and paper-based protocols but reported numerically different hypoglycemia and length-of-stay outcomes; however, these findings should be interpreted cautiously because the study was retrospective and not designed to establish causal superiority [19]. Sensitivity analysis showed that exclusion of Younis et al. eliminated heterogeneity in insulin infusion duration, indicating that this outcome was highly sensitive to individual study characteristics [18]. Ullal et al. reported faster resolution of metabolic acidosis and shorter hospital length of stay with Glucommander compared with standard care, but baseline DKA severity appeared imbalanced between groups [17].

A major limitation in interpreting time to DKA resolution is that resolution was not defined identically across studies. Ullal et al. defined resolution using blood glucose < 250 mg/dL and bicarbonate ≥ 18 mmol/L, Younis et al. used first anion gap ≤ 17, Martinez et al. used anion gap < 12 and measured time to transition from intravenous to subcutaneous insulin, Brown et al. reported time to anion gap closure ≤ 15 mEq/L, Groysman et al. used blood glucose < 200 mg/dL plus two biochemical criteria, and Fort et al. reported glycemic control as first blood glucose < 200 mg/dL and correction of acidosis as first serum bicarbonate > 16 mmol/L [17,18,19,20,21,22]. These differences mean that the pooled estimate combines related but non-identical clinical endpoints. Therefore, the primary outcome should be interpreted as a broad summary of reported resolution-related time outcomes rather than as a uniform measure of biochemical DKA resolution.

When compared with conventional insulin protocol groups, eGMSs showed no clear pooled difference in time to DKA resolution. Contrary to this pooled result, one retrospective study reported that treatment with Glucommander was associated with faster average time to DKA resolution compared with paper-based protocols, 12.9 versus 20.6 h [23]. In a multicenter trial in which ICU patients were randomized to continuous insulin infusion using Glucommander or a standard paper protocol, Glucommander showed a shorter time to target blood glucose, 4.8 ± 2.8 versus 7.8 ± 9.1 h [24]. Another study evaluating EndoTool reported that the average time to target blood glucose was 2.78 h in the standard group and 3.67 h in the EndoTool group, although this difference was not statistically significant [8]. Overall, although some individual studies suggested modest advantages with specific eGMS platforms, pooled analyses did not demonstrate a statistically or clinically meaningful improvement in efficacy outcomes. These isolated findings should be interpreted cautiously, given substantial heterogeneity and study-level risk of bias.

Subgroup analysis of adults versus children in time to DKA resolution did not identify a statistically significant difference between groups. However, this should not be interpreted as evidence that age does not modify treatment response, because the subgroup analysis was limited by a small number of studies and important physiological differences between adult and pediatric DKA populations, including cerebral edema risk, insulin sensitivity, and differences in fluid and electrolyte management.

Regarding length of stay, pooled analyses did not demonstrate statistically significant differences in ICU or hospital length of stay between eGMSs and conventional protocols, and sensitivity analyses showed that estimates were unstable across studies. Fort et al. contributed data on ICU length of stay, but its influence on heterogeneity limits the interpretation of this outcome [21]. Hospital length of stay also did not differ statistically between groups, but substantial heterogeneity and sensitivity to individual studies prevent a directional conclusion. Duration of insulin infusion showed inconsistent results across studies, and extreme heterogeneity prevents a reliable conclusion about whether eGMS shortens or prolongs treatment time. Therefore, the absence of statistical significance should not be interpreted as evidence of equivalence.

Consistent with these pooled findings, one retrospective study reported that use of Glucommander did not have a significant impact on median hospital length of stay compared with paper-based protocols, 3.7 versus 3.2 days [23]. Moreover, in a multicenter study comparing Glucommander with a standard paper insulin infusion algorithm, patients treated with Glucommander had similar mean ICU length of stay, 13.4 versus 8.5 days, and mean hospital length of stay, 17.5 versus 23.9 days [24]. However, evidence regarding EndoTool IV and DKA length of stay remains limited and should be interpreted cautiously. These mixed results highlight the need for well-designed multicenter trials to clarify the impact of eGMSs on length of stay and to identify factors influencing these outcomes.

The lack of a clear difference in length of stay may reflect variability in DKA severity at presentation, differences in discharge criteria, and the presence of comorbidities that extend hospitalization independent of glycemic management [25]. Sensitivity analyses indicated that single-study effects accounted for much of the observed heterogeneity, suggesting that institutional practices, discharge criteria, DKA severity, and case mix may affect length-of-stay outcomes independently of insulin delivery modality. In Ullal et al., patients were differentiated into mild, moderate, and severe DKA based on published diagnostic criteria; however, the groups appeared imbalanced, with more conventional protocol patients classified as having mild DKA and more eGMS patients classified as having severe DKA [17]. This imbalance makes comparisons difficult to interpret. Another consideration is the possible effect of closed versus open ICU systems. Several eGMS DKA studies evaluated patient populations managed in ICU or acute-care settings. The impact of ICU practice model on eGMS outcomes remains unclear and warrants further study.

The clearest observed safety signal was lower recorded hypoglycemia among patients managed with eGMSs. However, these estimates should not be interpreted as causal risk reductions because all included studies were non-randomized and vulnerable to confounding and outcome-detection bias. Several mechanisms could plausibly explain lower recorded hypoglycemia with eGMSs, including more frequent algorithmic dose adjustment, standardized insulin titration, automated prompts, reduced calculation errors, and earlier responses to falling glucose values. However, differential surveillance may also contribute to the observed effect. Conventional protocols may rely on manual glucose checks and documentation, whereas eGMS platforms may differ in automated recording, alerting, and monitoring workflows. If glucose measurements were more frequent or more completely documented in one group, apparent differences in hypoglycemia could partly reflect detection or documentation bias rather than true biological reduction in hypoglycemic events.

The severe hypoglycemia estimate was sensitive to the exclusion of Bouldin et al., suggesting that study-level differences influenced the pooled result. Bouldin et al. differed from the DKA-specific studies because they evaluated a broader inpatient insulin-management population that included cardiothoracic surgery, DKA, and non-DKA hyperglycemia patients, and because hypoglycemia was defined as blood glucose of 50–69 mg/dL, with severe hypoglycemia defined as blood glucose < 50 mg/dL [16]. The DKA subgroup was also small compared with the full study cohort. These features may partly explain why this study influenced the pooled estimate and reinforce that hypoglycemia findings should be interpreted as observational safety signals rather than causal evidence.

These results are consistent with previous evidence on digital and electronic glucose management technologies. A retrospective study comparing paper-based protocols with eGMSs in critically ill patients reported fewer hypoglycemic episodes after eGMS implementation, including a reduction in severe hypoglycemia, defined as blood glucose < 40 mg/dL, from 5.4% to 0.01% of treatment days [26]. In a study of bone marrow transplant patients, a population with substantial morbidity and mortality, the feasibility of eGMSs was prospectively evaluated. In this cohort of 21 instances of hyperglycemia managed with eGMSs, the rate of hypoglycemia, defined as blood glucose < 70 mg/dL, was 0.9%, and no patients experienced blood glucose levels less than 40 mg/dL [27]. Prior studies of digital diabetes management technologies have reported improved glycemic control and reduced hypoglycemia rates, particularly when interventions are supported by frequent monitoring and timely feedback from healthcare professionals [28]. However, potential workflow effects, including nursing workload, transcription errors, protocol adherence, and efficiency, were not consistently measured in the included DKA studies and therefore require dedicated evaluation before firm conclusions can be made.

The included studies evaluated different eGMS platforms, including Glucommander, EndoTool, Glytec, GlucoStabilizer, and Accu-Chek IV infusion. These platforms may differ in insulin-adjustment algorithms, glucose-monitoring intervals, input variables, alert systems, override procedures, and integration with institutional workflows. Therefore, the pooled results should not be interpreted as evidence that all eGMS platforms have equivalent efficacy or safety. Platform-specific subgroup analyses were limited by the small number of studies per platform, and future research should evaluate individual systems separately.

Current DKA management recommendations emphasize prompt fluid resuscitation, protocolized intravenous insulin therapy, potassium monitoring and replacement, frequent biochemical reassessment, identification of precipitating illness, and transition to subcutaneous insulin after resolution of ketoacidosis [9,10]. eGMS platforms may support insulin titration, but they do not replace these core components of DKA management. Therefore, eGMSs should be viewed as a potential insulin-dosing support tool rather than a comprehensive DKA management strategy.

### 4.1. Limitations

This review has several important limitations that substantially reduce certainty in the pooled estimates. First, all included studies were non-randomized, and all were judged to be at serious or critical overall risk of bias. The main risk-of-bias concerns were residual confounding, participant selection, deviations from intended interventions, missing data, and outcome-measurement bias. No included study was judged to be at low or moderate overall risk of bias.

Second, variability in DKA diagnostic criteria, DKA resolution definitions, hypoglycemia thresholds, glucose-monitoring frequency, and length-of-stay definitions limited comparability across studies. This heterogeneity likely reflects differences in eGMS platforms, insulin administration protocols, patient populations, clinical settings, and institutional practices.

Third, treatment assignment to eGMSs or conventional protocols was not randomized, and the included studies did not emulate a target trial with clearly aligned eligibility criteria, treatment assignment, time zero, follow-up, and analysis strategy. Therefore, residual confounding, confounding by indication, immortal time bias, and selection bias cannot be excluded. The pooled estimates should therefore be interpreted as associations rather than causal effects.

Fourth, protocol adherence, treatment deviations, crossovers, and whether analyses were conducted according to intention-to-treat, as-treated, or per-protocol principles were not consistently reported. Therefore, the meta-analysis primarily reflects available aggregate study-level estimates and cannot reliably account for non-adherence.

Fifth, hypoglycemia outcomes may be affected by detection bias. If glucose-monitoring frequency, automatic documentation, or charting completeness differed between eGMSs and conventional protocol groups, recorded hypoglycemia rates may not reflect true differences in event frequency.

Finally, the small number of studies per outcome limited the reliability of subgroup analyses, precluded formal meta-regression and publication-bias assessment, and reduced the precision of both conventional random-effects and HKSJ estimates.

### 4.2. Future Directions

Future research should address several methodological and clinical gaps. First, multicenter randomized controlled trials or well-designed target trial emulation studies are needed to estimate the causal effect of eGMSs compared with conventional protocols on DKA resolution, hypoglycemia, length of stay, and insulin infusion duration. Second, future studies should describe eGMS algorithms, glucose-monitoring intervals, alert systems, override procedures, and integration with local workflows in sufficient detail to support reproducibility. Third, a comprehensive evaluation framework should include cost-effectiveness, patient satisfaction, technology availability, nursing workload, transcription errors, and implementation feasibility. Such frameworks would support sustainable integration of eGMSs into routine care and inform policy decisions. Fourth, future studies should explore the differential efficacy and safety of eGMSs across patient subgroups defined by age, comorbidities, DKA severity, and baseline glycemic control. Fifth, the field of digital glycemic management is rapidly evolving, with continuous improvements in algorithms, monitoring devices, and integration with electronic health records. Some included studies may have used earlier-generation systems, which could limit applicability to newer eGMS platforms. Future studies should define time zero at insulin infusion initiation, standardize DKA diagnostic and resolution criteria, stratify by DKA severity and age group, report protocol adherence, ensure comparable glucose-monitoring intensity between groups, and conduct platform-specific analyses.

## 5. Conclusions

In conclusion, this systematic review and meta-analysis of non-randomized studies found no clear evidence that eGMS-guided insulin infusion improves time to DKA resolution, ICU length of stay, hospital length of stay, or duration of insulin infusion compared with conventional insulin infusion protocols. eGMSs were associated with lower recorded rates of mild and severe hypoglycemia; however, this finding should be interpreted cautiously because all included studies were at serious or critical risk of bias, several outcomes showed substantial heterogeneity, and differential glucose-monitoring or documentation practices may have influenced hypoglycemia detection. The current evidence should therefore be considered hypothesis-generating rather than definitive. High-quality randomized trials and target trial emulation studies are needed before recommending widespread adoption of eGMSs over conventional protocols for DKA management.

## Figures and Tables

**Table 1:** Population, Intervention, Comparison and Outcome (PICO) table.

Population	Pediatric and adult patients diagnosed with DKA
Intervention	Computer-guided IV insulin infusion algorithms (Glucommander, Glytec, EndoTool, GlucoStabilizer, Accu-Chek IV infusion)
Control	Conventional paper-based or column-based IV insulin infusion
Outcome	Time to resolution of DKA

**Table 2:** DKA diagnostic and resolution definitions across included studies.

Study	Population Included	DKA Diagnostic Criteria Reported by Study	DKA Severity Criteria Reported	DKA Resolution Definition Used in Study	Hypoglycemia Threshold	Mixed DKA/HHS Population?	Included in Primary Time-to-Resolution Analysis?	Notes for Interpretation
Bouldin et al., 2021 [16]	Adults ≥ 18 years treated on two pilot units; full cohort included CT surgery, DKA, and non-DKA hyperglycemia patients; DKA subgroup: 22 eGMSs and 33 comparison patients	NR	NR	NR for DKA resolution; primary efficacy endpoint was percent of POC BG readings in target glycemic range after target was reached	Hypoglycemia: BG 50–69 mg/dL; severe hypoglycemia: BG < 50 mg/dL	No HHS subgroup reported; cohort included DKA and non-DKA hyperglycemia/CT surgery patients	No	Study was not DKA-only and did not assess time to DKA resolution; target range for DKA/non-DKA hyperglycemia patients was 70–200 mg/dL
Ullal et al., 2018 [17]	Adults > 18 years admitted to ICU or step-down floors with DKA	DKA based on ICD-9 codes 250.10, 250.11, 250.12, and 250.13, corroborated by BG > 200 mg/dL, bicarbonate < 18 mmol/L, and anion gap > 12 mEq/L	Mild, moderate, and severe DKA categories were used based on previously published DKA diagnostic criteria	Time to DKA resolution defined as time to achieve BG < 250 mg/dL and bicarbonate ≥ 18 mmol/L	Hypoglycemia: BG < 70 mg/dL; severe hypoglycemia: BG ≤ 40 mg/dL	No	Yes	Discontinuation of insulin infusion was based on clinician judgment, including resolution of hyperglycemia and acidosis with anion-gap closure
Younis et al., 2020 [18]	Adults > 18 years with type 1 or type 2 diabetes admitted directly from ED to ICU with DKA and treated with insulin infusion within 4 h	Glucose > 250 mg/dL, bicarbonate < 18 mEq/L, and urine or blood ketones	NR	Time to first anion gap ≤ 17 minus insulin infusion initiation time	Mild hypoglycemia: BG < 80 mg/dL; severe hypoglycemia: BG < 50 mg/dL	No; HHS excluded	Yes	Basic metabolic panels checked every 4 h; POC BG checked hourly in paper group, while GM determined timing in computer-based group
Martinez et al., 2023 [19]	Adults ≥ 18 years admitted with primary diagnosis of DKA	BG > 250 mg/dL, positive serum/urine ketones, and anion gap > 15	NR	DKA resolution defined as anion gap < 12; primary outcome was time from DKA protocol initiation to transition from IV to subcutaneous insulin	Hypoglycemia: BG < 70 mg/dL; severe hypoglycemia: BG < 40 mg/dL	No; HHS excluded	Yes	Manual eGMS dose adjustments were possible; paper-based protocol adherence/deviation data could not be obtained
Brown et al., 2023 [20]	Patients on insulin infusion treated in ED or ICU for DKA or HHS; DKA subgroup: 69 eGMSs and 70 PBP patients; HHS subgroup: 15 eGMSs and 14 PBP patients	NR	NR	Time to anion gap closing, defined as anion gap ≤ 15 mEq/L	Hypoglycemia: BG < 70 mg/dL; severe hypoglycemia threshold NR	Yes; DKA and HHS were both included, with subgroup results reported	Yes, if DKA-specific subgroup data were used; otherwise, mixed DKA/HHS data require caution	DKA and HHS were reported as subgroups for several outcomes; BG checks within protocol timing were 80.7% with eGMSs vs. 52.6% with PBP
Fort et al., 2009 [21]	Children aged 2–18 years with new-onset or existing type 1 diabetes admitted to PICU with DKA	NR	NR	Glycemic control defined as first BG < 200 mg/dL; correction of acidosis defined as first serum bicarbonate > 16 mmol/L	Inadvertent hypoglycemia: BG < 40 mg/dL	No	Yes	Pediatric-only study; starting analysis values were blood glucose and bicarbonate at presentation, including children transported after receiving insulin/fluids
Groysman et al., 2020 [22]	Nonpregnant adults ≥ 18 years admitted with DKA	ADA criteria: BG 250 mg/dL, arterial pH ≤ 7.30, serum bicarbonate ≤ 18 mEq/L, anion gap > 10 mEq/L, and positive β-hydroxybutyrate or acetone	NR	BG < 200 mg/dL plus 2 of the following: pH > 7.3, bicarbonate ≥ 15 mEq/L, and anion gap ≤ 12 mEq/L	Hypoglycemia: BG < 70 mg/dL; clinically significant hypoglycemia: BG < 54 mg/dL; severe hypoglycemia: BG < 40 mg/dL	No; HHS excluded	Yes	Excluded HHS, SGLT2 inhibitor-associated euglycemic DKA, ESRD, and patients managed by both insulin methods during the same stay

BG, blood glucose; DKA, diabetic ketoacidosis; ED, emergency department; eGMS, electronic glycemic management system; GM, Glucommander; HHS, hyperosmolar hyperglycemic state; ICU, intensive care unit; IV, intravenous; NR, not reported; PBP, paper-based protocol; PICU, pediatric intensive care unit; POC, point of care.

**Table 3:** Secondary Outcomes.

Secondary Outcomes
Incidence of mild hypoglycemia
Incidence of severe hypoglycemia
Intensive Care Unit (ICU) length of stay
Hospital length of stay
Duration of insulin infusion

**Table 4:** GRADE certainty of evidence profile.

Outcome	No. of Studies	Study Design	Risk of Bias	Inconsistency	Indirectness	Imprecision	Publication Bias	Overall Certainty	Explanation
Time to DKA resolution	6	Non-randomized observational studies	Very serious	Serious	Serious	Serious	Not assessed	Very low	All studies were non-randomized with serious-to-critical risk of bias; definitions of DKA resolution varied across studies; heterogeneity was substantial.
ICU length of stay	3	Non-randomized observational studies	Very serious	Serious	Serious	Serious	Not assessed	Very low	Few studies contributed data; clinical settings differed; leave-one-out analysis identified Fort et al. as influential.
Hospital length of stay	5	Non-randomized observational studies	Very serious	Very serious	Serious	Serious	Not assessed	Very low	Heterogeneity was very high; excluding Ullal et al. changed the direction of the point estimate, so no directional conclusion should be inferred.
Duration of insulin infusion	3	Non-randomized observational studies	Very serious	Very serious	Serious	Serious	Not assessed	Very low	Heterogeneity was extreme; excluding Younis et al. changed direction and significance, so this outcome is best interpreted narratively.
Mild hypoglycemia	6	Non-randomized observational studies	Very serious	Very serious	Serious	Serious	Not assessed	Very low	Lower recorded hypoglycemia was observed with eGMS, but heterogeneity, residual confounding, variable definitions, and detection bias were major concerns.
Severe hypoglycemia	6	Non-randomized observational studies	Very serious	Serious	Serious	Serious	Not assessed	Very low	Lower recorded severe hypoglycemia was observed with eGMS, but the pooled estimate was sensitive to exclusion of Bouldin et al., and detection/documentation bias was possible.

GRADE, Grading of Recommendations Assessment, Development and Evaluation; DKA, diabetic ketoacidosis; eGMS, electronic glycemic management system; ICU, intensive care unit. Certainty was rated very low across outcomes because all included studies were non-randomized and had serious-to-critical risk of bias, with additional concerns regarding inconsistency, indirectness, and imprecision.

**Table 5:** Inclusion and exclusion criteria.

Inclusion Criteria	Exclusion Criteria
Studies involving adult or pediatric patients diagnosed with DKA according to study-defined diagnostic criteria, with diagnostic thresholds extracted where available	Studies including only HHS, or mixed DKA/HHS cohorts without separable DKA data or without treatment under a DKA insulin infusion protocol
Comparative studies evaluating computer-guided or electronic insulin infusion systems (e.g., Glucommander, EndoTool, GlucoStabilizer, etc.)	Studies evaluating subcutaneous insulin therapy only without IV insulin infusion
Studies comparing electronic insulin systems with conventional (paper-based or provider-directed) insulin infusion protocols	Animal studies, simulation studies, or in vitro experiments
Observational cohort studies (prospective or retrospective)	Conference abstracts without full-text data or insufficient outcome reporting
Studies reporting at least one relevant outcome (e.g., time to DKA resolution, hypoglycemia, ICU or hospital length of stay, duration of insulin infusion)	Non-peer-reviewed articles or narrative reviews
Studies published in the English language	

Studies enrolling mixed DKA/HHS populations were included only when DKA-specific data were reported separately or when patients were managed under a DKA insulin infusion protocol. Mixed DKA/HHS cohorts were considered a source of indirectness and clinical heterogeneity.

**Table 6:** Characteristics of eGMS platforms and comparator protocols across included studies.

Study	eGMS Platform	Algorithm/Input details Reported	Glucose-Monitoring Interval	Alerts/Prompts Reported?	EHR/EMR Integration Reported?	Clinical Setting	Comparator Protocol	Platform-Related Heterogeneity Issue
Bouldin et al., 2021 [16]	Glucommander	eGMS-directed IV and SQ insulin adjustments based on BG rate of change and instructed when next BG should be checked	BG checks performed at intervals directed by eGMS or Lien–Spratt nomogram	Yes; eGMS-directed timing of next BG check	NR	Community hospital pilot units: critical care and telemetry	Lien–Spratt nomogram or Bell–Bass nomogram, followed by provider-managed SQ insulin	Broader cohort included CT surgery, DKA, and non-DKA hyperglycemia; DKA subgroup was small
Ullal et al., 2018 [17]	Glucommander, Glytec	Uses multiplier/insulin sensitivity factor; formula: insulin/hour = multiplier × (BG − 60); multiplier automatically adjusted based on glucose pattern and insulin response	GM could recommend 30-min checks during rapid glucose decline; conventional protocols used site-specific practice	Yes; GM recommended insulin dose and next BG check; alert for high anion gap to prevent premature discontinuation	Yes; GM integrated with EMR/lab interface, with EPIC used at most hospitals	ICU or step-down floors across 34 institutions	Standard column-based or paper protocols based on protocols such as Yale or Leuven	Multicenter study with site-specific target BG ranges and conventional protocols
Younis et al., 2020 [18]	Glucommander licensed by Glytec	Inputs included height, weight, BG, HbA1c, carbohydrate consumption, target glucose, and multiplier/insulin sensitivity factor; institutional multiplier 0.01	BMP every 4 h; POC BG hourly in paper group; GM determined BG-check timing in computer group	Yes; GM recommended insulin infusion rate and next BG-check timing	GM installed on ICU computers; EPIC used for data extraction	ICU in two-hospital health system	Paper-based insulin infusion algorithm created by institutional endocrinologists using ADA guidance	Differential glucose-monitoring timing between groups may affect hypoglycemia detection
Martinez et al., 2023 [19]	Glytec eGMS	Initial multiplier 0.01 units/kg/h; target BG 140–180 mg/dL; multiplier adapted using hourly BG data points; manual entry adjustments permitted	Hourly BG data points reported in eGMS	NR	Yes; eGMS integrated into EMR	ICU at large university-based teaching hospital	Institution-specific paper-based DKA protocol; insulin 0.1 units/kg/h titrated by 0.05 units/kg/h until BG 150–250 mg/dL	Manual dose adjustments possible; paper protocol not fully documented in EMR
Brown et al., 2023 [20]	EndoTool	Uses patient-specific factors, including height, weight, diabetes status, renal function, steroid dosing, insulin response, and estimated residual extracellular insulin	PBP checks ranged 30–90 min; eGMS determined next BG check	Yes; alarm/alert for next BG check and transition to SQ insulin	NR	ED and ICU in VA hospital	Weight-based paper-based dosing nomogram/PBP	Mixed DKA/HHS population; protocol-timing adherence differed between groups
Fort et al., 2009 [21]	Glucommander	Physician-selected glucose target range and weight-based multiplier; target BG 80–140 mg/dL; insulin dose/hour = (BG − 60) × multiplier; pediatric multiplier = weight × 0.0002	Algorithm adjusted every hour based on serial bedside glucose values; required hourly manual finger-sticks	Yes; recommended insulin infusion rate and interval to next glucose measurement	NR	PICU	Two-bag manually titrated insulin infusion	Pediatric-only algorithm modification; requires correct target/multiplier entry
Groysman et al., 2020 [22]	GlucoStabilizer	Initial multiplier 0.01; target range 140–200 mg/dL; no IV insulin bolus; multiplier models insulin sensitivity and changes with BG response	Metabolic profile measured every 4–6 h; BG monitoring interval NR	Yes; GlucoStabilizer calculates insulin dose and alerts nurses when to measure BG	EMR interoperability was a deciding factor in software selection	Hospital DKA management before/after software implementation	ADA protocol-directed provider-guided insulin adjustment; initial insulin 0.1 units/kg/h without bolus	Staff instructed to override software recommendation of 0 units/h if acidosis persisted

BG, blood glucose; BMP, basic metabolic panel; DKA, diabetic ketoacidosis; ED, emergency department; eGMS, electronic glycemic management system; EHR, electronic health record; EMR, electronic medical record; ICU, intensive care unit; IV, intravenous; NR, not reported; PBP, paper-based protocol; PICU, pediatric intensive care unit; POC, point of care; SQ, subcutaneous.

**Table 7:** Protocol adherence, deviations, and analysis approach across included studies.

Study	Intended Insulin Protocol Clearly Described?	Protocol Adherence Rate Reported?	Protocol Deviations or Overrides Reported?	Crossovers or Mixed Protocol Exposure Reported?	Glucose-Monitoring Adherence Reported?	Analysis Approach Reported	Adjustment for Non-Adherence?	Interpretation
Bouldin et al., 2021 [16]	Yes	NR	Yes; due to data-system limitations, patients in either group may have received one-time insulin doses outside the intended nomogram/eGMS	Yes/partial; eGMS patients were excluded if they did not use both IV and SQ eGMS modules; comparison patients could transfer floors	NR	Retrospective cohort; full-sample and indication subgroup analyses	No	Protocol contamination possible because one-time provider insulin orders outside the intended system may have occurred
Ullal et al., 2018 [17]	Yes	NR	Planned EMR downtime interruptions were excluded; conventional protocols varied by site	No contemporaneous within-site use of GM and paper protocols reported	NR	Retrospective multicenter comparison; two-sample *t*-tests not assuming equal variance	No	Conventional arm was heterogeneous because protocols differed across sites
Younis et al., 2020 [18]	Yes	NR	NR	Grouping by pre-/post-implementation period; no crossover described	BMP every 4 h; POC BG hourly in paper group; BG timing determined by GM in computer group	Retrospective pre-/post-analysis; *t*-test, Wilcoxon rank-sum, chi-square; multiple linear regression and logistic regression also reported	No for pooled meta-analysis; adjusted analyses were reported in study but not consistently available across studies	Differential BG monitoring frequency may affect hypoglycemia detection
Martinez et al., 2023 [19]	Yes	NR	Yes; manual entry adjustments of eGMS multipliers occurred; impact unclear	No eGMS discontinuation/re-entry reported	NR	Retrospective pre-/post-analysis; *t*-test or Mann–Whitney U; chi-square/Fisher’s exact test	No	Paper protocol was not fully documented in EMR, so adherence/deviation data could not be obtained
Brown et al., 2023 [20]	Yes	NR for full protocol adherence	NR	NR	Yes; BG checks within protocol timing were 80.7% with eGMSs vs. 52.6% with PBP	Retrospective chart review; statistical testing reported for outcomes	No	eGMS improved timing adherence, but broader protocol adherence and deviations were not fully reported
Fort et al., 2009 [21]	Yes	NR	NR	Six children had multiple DKA admissions and were managed with both methods across different admissions, not during same admission	Hourly manual finger-sticks required with Glucommander	Retrospective chart review; measures of central tendency	No	Protocol adherence not quantified; correct initial data entry was noted as important
Groysman et al., 2020 [22]	Yes	NR	Yes; staff were instructed to override GlucoStabilizer recommendation of 0 units/h and continue IV insulin at 0.3 units/h if acidosis persisted	Patients managed by both methods during the same hospital stay were excluded	Metabolic profile every 4–6 h; BG-monitoring adherence NR	Retrospective quasi-experimental before/after analysis; Wilcoxon rank-sum, *t*-test, Fisher’s exact test; multivariable model for log-transformed insulin units	No adjustment for DKA-resolution confounders; multivariable model only reported for insulin units	Study explicitly states that it did not adjust for factors that could affect time to DKA resolution

BG, blood glucose; BMP, basic metabolic panel; DKA, diabetic ketoacidosis; ED, emergency department; eGMS, electronic glycemic management system; EHR, electronic health record; EMR, electronic medical record; ICU, intensive care unit; IV, intravenous; NR, not reported; PBP, paper-based protocol; PICU, pediatric intensive care unit; POC, point of care; SQ, subcutaneous.

**Table 8:** Baseline characteristics of included studies.

Study	Bouldin et al., 2021 [16]	Ullal et al., 2018 [17]	Younis et al., 2020 [18]	Martinez et al., 2023 [19]	Brown et al., 2023 [20]	Fort et al., 2009 [21]	Groysman, 2020 [22]
Population	Hospitalized patients	Patients ≥ 18 yrs admitted to ICU or step-down floors with DKA	Adults (>18 yr) with type 1 or 2 DM admitted to the ICU with DKA	Patients admitted for management of DKA	Patients with DKA or HHS admitted to the ICU or ED	Type 1 DM patients admitted for DKA	Patients with DKA
Intervention (I)	eGlycemic management system	Glucommander	Glucommander	Glytec	EndoTool	Accu-Chek IV infusion	GlucoStabilizer
Control (C)	nomogram-driven IV insulin therapy followed by provider-managed basal-bolus S/C insulin	Conventional protocol	Conventional insulin infusion protocol	MDI (basal bolus) protocol	Paper-based protocol	2006 institutional DKA protocol	Physician-driven insulin protocol
Study Design	Retrospective cohort	Retrospective cohort	Retrospective pre-/post-cohort study	Retrospective analysis	Single-center retrospective study	Retrospective chart review	Single-center retrospective study
Sample size, n (I/C)	110/108	1750/915	373/247	84/133	84/84	65/22	77/69
Female, n (%) (I/C)	32 (29.1%)/46 (57.4%)	901 (51.5%)/490 (53.6%)	195 (52%)/141 (57%)	53 (63.1%)/74 (55.6%)	NR	35 (54%)/14 (63%)	36 (46.7%)/31 (44.9%)
Mean age ± SD in years (I/C)	63.1 ± 12.4/60 ± 14.8	44.3 ± 17.8/49.6 ± 23.2	43.8 ± 20.06/42.21 ± 26.24	45.9 ± 16.1/41.9 ± 16.7	62.95 ± 11.44/60.9 ± 11.44	12/12	47.3 ± 8.0/44.0 ± 7.5
BMI (kg/m^2^) (Mean ± SD)	NR	26.8 ± 11.9/26.8 ± 10.9	27 ± 6/26.8 ± 7	27.5 ± 9.3/25.7 ± 6	29.9 ± 6.61/28.63 ± 6.61	NA	NA
Baseline Blood Glucose, mg/dL (I/C) (Mean ± SD)	NR	598 ± 255/425 ± 249	579 ± 198/551 ± 196	614 ± 248/557 ± 248	NR	464/410	543 ± 60/539.3 ± 66.5
Bicarbonate (mmol/L, equivalent to mEq/L) (I/C) (Mean ± SD)	NR	11 ± 4.5/14 ± 4.1	9 ± 5.93/10.95 ± 5.45	9.4 ± 4.1/10.0 ± 4.1	NR	11/9	11.3 ± 2.1/11.6 ± 1.5
pH (I/C) (Mean ± SD)	NR	7.2 ± 2.5/7.2 ± 1.9	NR	7.13 ± 0.16/7.18 ± 0.39	NR	NR	7.2 ± 0.04/7.2 ± 0.05
K^+^ (Mean ± SD)	NA	NA	NA	4.9 ± 1.1/5.1 ± 3.2	NR	NR	NR
HbA1c (%)	NR	10.8 ± 5.3/9.3 ± 5.0	11.6 ± 2.8/11.4 ± 2.4	12.4 ± 2.8/12.3 ± 2.5	11.8 ± 3.03/11.8 ± 3.03	NR	11.3 ± 2.1/11.6 ± 1.5

APACHE II, Acute Physiology and Chronic Health Evaluation II; BMI, body mass index; DKA, diabetic ketoacidosis; DM, diabetes mellitus; ED, emergency department; HbA1c, glycated hemoglobin; HHS, hyperosmolar hyperglycemic state; ICU, intensive care unit; IV, intravenous; MDI, multiple daily insulin injections; NA, not applicable; NR, not reported; S/C, subcutaneous.

## Data Availability

The original data presented in this study are openly available at https://zenodo.org/ at https://doi.org/10.5281/zenodo.18674798.

## Supplementary Materials

The following supporting information can be downloaded at https://www.mdpi.com/article/10.3390/medicina62071287/s1: Table S1: MOOSE (Meta-analyses Of Observational Studies in Epidemiology) checklist; Table S2: Comprehensive search strategies and results across electronic databases for studies comparing computer-guided and standard insulin infusion protocols in diabetic ketoacidosis and hyperglycemic hyperosmolar states.
