# Supplementary material for: Electronic Glycemic Management Systems Versus Conventional Insulin Infusion Protocols in Diabetic Ketoacidosis: A Systematic Review and Meta-Analysis of Non-Randomized Studies
Source: Medicina (Kaunas). 2026 Jul 3;62(7):1287. doi: 10.3390/medicina62071287 (PMC13413941; doi:10.3390/medicina62071287)
Supplement: Supplementary file 1 [file medicina-62-01287-s001.zip › Supplementary Table S1.pdf]

Supplementary Table S1: Comprehensive Search Strategies and Results Across Electronic Databases for Studies Comparing Computer-Guided and Standard Insulin Infusion Protocols in Diabetic Ketoacidosis and Hyperglycemic Hyperosmolar States

Database	Number of articles	Search strategy
PubMed	94	((Diabetic ketoacidosis) OR (DKA) OR (HHS) OR (Hyperosmolar Non-Ketotic State) OR (HONK) OR (Hyperglycemic Hyperosmolar State)) AND ((Computer guided insulin infusion algorithm) OR (Glucommander) OR (Computer based insulin infusion algorithm) OR (ELECTRONIC-BASED DKA PROTOCOL) OR (Glucostabilizer) OR (electronic glucose management system) OR (eGMS)) AND ((manually titrated infusion) OR (continuous insulin infusion) OR (GM) OR (standard insulin infusion) OR (Paper based insulin infusion algorithm) OR (provider-guided insulin dose adjustment))
Embase	194	('diabetic ketoacidosis'/exp OR 'diabetic ketoacidosis' OR (('diabetic'/exp OR diabetic) AND ('ketoacidosis'/exp OR ketoacidosis)) OR dka OR hhs OR 'hyperosmolar non-ketotic state' OR (hyperosmolar AND 'non ketotic' AND ('state'/exp OR state)) OR honk OR 'hyperglycemic hyperosmolar state'/exp OR 'hyperglycemic hyperosmolar state' OR (hyperglycemic AND hyperosmolar AND ('state'/exp OR state))) AND ('computer guided insulin infusion algorithm' OR (('computer'/exp OR computer) AND guided AND ('insulin'/exp OR insulin) AND ('infusion'/exp OR infusion) AND ('algorithm'/exp OR algorithm)) OR 'glucommander'/exp OR glucommander OR 'computer based insulin infusion algorithm' OR (('computer'/exp OR computer) AND based AND ('insulin'/exp OR insulin) AND ('infusion'/exp OR infusion) AND ('algorithm'/exp OR algorithm)) OR 'electronic-based dka protocol' OR

		('electronic based' AND dka AND ('protocol'/exp OR protocol)) OR glucostabilizer OR 'electronic glucose management system' OR (electronic AND ('glucose'/exp OR glucose) AND ('management'/exp OR management) AND system) OR egms) AND ('manually titrated infusion' OR (manually AND titrated AND ('infusion'/exp OR infusion)) OR 'continuous insulin infusion'/exp OR 'continuous insulin infusion' OR (continuous AND ('insulin'/exp OR insulin) AND ('infusion'/exp OR infusion)) OR 'gm'/exp OR gm OR 'standard insulin infusion' OR (('standard'/exp OR standard) AND ('insulin'/exp OR insulin) AND ('infusion'/exp OR infusion)) OR 'paper based insulin infusion algorithm' OR (('paper'/exp OR paper) AND based AND ('insulin'/exp OR insulin) AND ('infusion'/exp OR infusion) AND ('algorithm'/exp OR algorithm)) OR 'provider-guided insulin dose adjustment' OR ('provider guided' AND ('insulin'/exp OR insulin) AND ('dose'/exp OR dose) AND ('adjustment'/exp OR adjustment)))
Scopus	677	('diabetic ketoacidosis'/exp OR 'diabetic ketoacidosis' OR (('diabetic'/exp OR diabetic) AND ('ketoacidosis'/exp OR ketoacidosis)) OR dka OR hhs OR 'hyperosmolar non-ketotic state' OR (hyperosmolar AND 'non ketotic' AND ('state'/exp OR state)) OR honk OR 'hyperglycemic hyperosmolar state'/exp OR 'hyperglycemic hyperosmolar state' OR (hyperglycemic AND hyperosmolar AND ('state'/exp OR state))) AND ('computer guided insulin infusion algorithm' OR (('computer'/exp OR computer) AND guided AND ('insulin'/exp OR insulin) AND ('infusion'/exp OR infusion) AND ('algorithm'/exp OR algorithm)) OR

		'glucommander'/exp OR glucommander OR 'computer based insulin infusion algorithm' OR (('computer'/exp OR computer) AND based AND ('insulin'/exp OR insulin) AND ('infusion'/exp OR infusion) AND ('algorithm'/exp OR algorithm)) OR 'electronic-based dka protocol' OR ('electronic based' AND dka AND ('protocol'/exp OR protocol)) OR glucostabilizer OR 'electronic glucose management system' OR (electronic AND ('glucose'/exp OR glucose) AND ('management'/exp OR management) AND system) OR egms) AND ('manually titrated infusion' OR (manually AND titrated AND ('infusion'/exp OR infusion)) OR 'continuous insulin infusion'/exp OR 'continuous insulin infusion' OR (continuous AND ('insulin'/exp OR insulin) AND ('infusion'/exp OR infusion)) OR 'gm'/exp OR gm OR 'standard insulin infusion' OR (('standard'/exp OR standard) AND ('insulin'/exp OR insulin) AND ('infusion'/exp OR infusion)) OR 'paper based insulin infusion algorithm' OR (('paper'/exp OR paper) AND based AND ('insulin'/exp OR insulin) AND ('infusion'/exp OR infusion) AND ('algorithm'/exp OR algorithm)) OR 'provider-guided insulin dose adjustment' OR ('provider guided' AND ('insulin'/exp OR insulin) AND ('dose'/exp OR dose) AND ('adjustment'/exp OR adjustment)))
Cochrane Central Register of Controlled Trials	0	((Diabetic ketoacidosis) OR (DKA) OR (HHS) OR (Hyperosmolar Non-Ketotic State) OR (HONK) OR (Hyperglycemic Hyperosmolar State)) AND ((Computer guided insulin infusion algorithm) OR (Glucommander) OR (Computer based insulin infusion algorithm) OR (ELECTRONIC-BASED DKA

		PROTOCOL) OR (Glucostabilizer) OR (electronic glucose management system) OR (eGMS)) AND ((manually titrated infusion) OR (continuous insulin infusion) OR (GM) OR (standard insulin infusion) OR (Paper based insulin infusion algorithm) OR (provider-guided insulin dose adjustment))
Clinicaltrails.gov	1	((Diabetic ketoacidosis) OR (DKA) OR (HHS) OR (Hyperosmolar Non-Ketotic State) OR (HONK) OR (Hyperglycemic Hyperosmolar State)) AND ((Computer guided insulin infusion algorithm) OR (Glucommander) OR (Computer based insulin infusion algorithm) OR (ELECTRONIC-BASED DKA PROTOCOL) OR (Glucostabilizer) OR (electronic glucose management system) OR (eGMS)) AND ((manually titrated infusion) OR (continuous insulin infusion) OR (GM) OR (standard insulin infusion) OR (Paper based insulin infusion algorithm) OR (provider-guided insulin dose adjustment))
